# Atlanta Academy of Medicine

**Published:** 1877-04

**Authors:** 


### ATLANTA ACADEMY OF MEDICINE.

O. R. FORD, M.D., REPORTER.

ATLANTA, February 27, 1877.

The President, Dr. V. H. Taliaferro, in the chair.

Dr. Calhoun called the attention of the Academy to a case of sympathetic ophthalmia, and the treatment, which consisted in the enucleation of the diseased eye. Also to the length of time after the injury before the appearance of the sympathetic inflammation in the sound eye. The case was one of an old lady, æt. 63. Thirty-three years ago she received an injury in the left eye, through the ciliary bodies, followed by a loss of vision. One year ago vision began to fail in the sound eye, attended by all the prodromic symptoms of sympathetic ophthalmia. The points of special interest are the great length of time after the injury before the appearance of the sympathetic inflammation, namely thirty-three years; the rapid recovery and the rapid extension of the disease; also the remarkable time required for anæsthesia, only five minutes being necessary. Inflammation of the ciliary bodies, either idiopathic or traumatic, is the most frequent cause of sympathetic ophthalmia. When there is an injury through the ciliary bodies, and the vision is lost, it is better to enucleate at once, because, through the resulting irritation, sympathetic inflammation almost invariably follows.

Dr. Baird wished to know what were the evidences of a wound in the ciliary bodies.

Dr. Calhoun explained the position of the ciliary bodies as being immediately behind the corneo-sclerotic junction. Injury to these, when sufficient to destroy the vision, will almost invariably cause sympathetic inflammation in the sound eye. This is according to his experience, and reported statistics. Pain is felt on pressure, and there is usually a corresponding painful point in the other eye after the outburst of the sympathetic inflammation.

Dr. Baird thought there ought to be some undoubted evidence of an injury to the ciliary bodies before enucleation, and inquired as to whether this condition might not occur without a wound; may it not be accidental.

Dr. Calhoun said that his experience convinced him that an injury to the ciliary bodies was the cause of sympathetic inflammation in the majority of cases. When the body is injured it rarely recovers; chronic inflammation supervenes, and any exciting cause suddenly kindles it up. If an injury had ever existed there would be no difficulty in tracing the disease back to it.

Academy adjourned.

---

ATLANTA, March 6, 1877.

The President, Dr. Taliaferro, in the chair.

Dr. Simpson has recently had some experience with salicylic acid in the treatment of articular rheumatism. He reported a case in which he had given the remedy in the dose of ten grains, repeated every two hours, with marked success. The patient was free from suffering and convalescent in ten days from the attack. The only difficulty experienced in the action of the remedy was that of nausea. He called for the experience of those members who had been in the habit of using it.

Dr. Baird had never used salicylic acid in rheumatism, but had read reports of its successful employment in the treatment of this disease. It is claimed, however, that salicine is equally efficacious, and does not cause the gastric irritation that frequently follows the administration of salicylic acid.

Dr. J. G. Westmoreland said that the treatment of rheumatism with salicylic acid was in line with the quinine treatment, which he had pursued fo. twenty years. It is known, said he, that quinine, salicine, and salicylic acid are invigorators of the spinal cord, and as rheumatism has been found to yield under their action, he finds confirmation of his theory, that the disease essentially depends upon disturbance of this nervous centre.

Dr. Baird said he inferred from Dr. Westmoreland's remarks that he located the seat of the disease in the spinal cord, and asked for his ideas of its pathology.

Dr. Westmoreland: I cannot give the condition of the spinal cord, but I know that it is disturbed. Aside from this the symptoms can be explained only on the idea of nervous derangement. The migratory character of the local manifestations indicates their nervous origin. Pain, soreness and swelling in one joint to-night, may have disappeared by to-morrow, being transferred to some joint on the opposite side of the body. If this local manifestation was the direct result of irritating or poisonous substances in the blood, as many suppose, this sudden transfer would not occur. It is not therefore ordinary inflammation progressing to suppuration. An abscess is never found as the result of true rheumatic inflammation. He has been accustomed to treat this disease with quinine and opium, which he finds sufficient, usually, to effect a cure. Where it fails, he applies a blister to the spinal column. He thought that malaria affected the same part that is disturbed in the production of rheumatism; but in both of the diseases the invigorating effect of quinine or salicine is generally salutary, particularly when used in connection with opium and counter-irritation over the spine. Salicine had failed in his hands, he thinks on account of the smallness of the dose; it requiring at least double the quantity in which quinine is used.

Dr. Todd acquiesced with Dr. Westmoreland in regard to the beneficial results obtained by the application of the blister over the spinal cord. He thinks it is the means of effecting a cure in most instances. He also uses opiates to relieve pain, and administers alkalies to change the acid condition of the urine.

Dr. Taliaferro alluded to the importance of giving opium and quinine in connection. He is of the opinion that the efficacy of the two remedies is greatly increased when given together.

---

ATLANTA, March 13, 1877.

The President, Dr. Taliaferro, in the chair.

Dr. McIntosh presented a pathological specimen, consisting of the kidneys, urethra, bladder and prostate gland. The specimen was obtained from a man forty-five years old, and shows enlargement of the middle lobe of the prostate, which led to the patient's death by obstructing the passage of urine. The subject had been troubled for five years with difficult micturition and other symptoms of cystitis and prostatitis. Introduction of the catheter being difficult, prostatic enlargement was suspected, and the daily evacuation of a large quantity of very offensive urine and pus with the catheter, and injection of carbolic acid water was continued for five weeks, when worn down by irritation, the patient died; diarrhœa having set in three days before. Subsultus and stupor toward the termination led to suspicion of uræmic poison as a factor in the cause of death. The use of nourishing diet, with tonics, was had from the beginning, and after two weeks, improvement was manifest, the pus being less abundant in urine, and the circulation, which had numbered 112, was reduced to 90. Post-mortem examination revealed the following: Bowels red and congested; bladder inflamed, hypertrophied, and fasciculated; right kidney much contracted; cortical substance very thin; calices and pelvis much dilated; left kidney normal in size. The lateral lobes of prostate were very little enlarged, but the middle lobe projects upward, in almost a globular form, nearly half an inch in diameter, so as to obstruct the urethral canal.

The retained urine doubtless, by its mechanical distention and its decomposition, caused the inflammation and hypertrophy of the bladder; and by the reflex pressure, the dilatation and disintegration of the kidney. The injuries to the kidneys lessened the secreting capacity of the organs, and led to the uræmic poisoning. The great desideratum in the treatment of such cases, therefore, is to keep the bladder emptied regularly and perfectly with the catheter.

Dr. Atlee, of Philadelphia, uses fluid extract ergot in free doses, in addition to the regular evacuation of the urine, and, he thinks, with benefit to the patient.

It was suggested by Guthrie to cut through the perineum, make a permanent fistula, and in this way drain the bladder. This has been done four times by American surgeons. Dr. Sims has made openings into the female bladder, for the relief of obstinate cystitis, which did not yield to other means, and with success. Cystotomy, in one of the four male cases, has been of great benefit. In the three others the result is not known. Older surgeons were in the habit of crushing median prostatic enlargement, through the urethra, but without encouraging success.

I think that the removal of such obstructing portion of the prostate by an opening through the perineum, as in the lateral operation for stone, would be the most rational and promising course, where milder means fail to give relief. Such operation would not necessarily lead to disastrous effects, as is proven by the loss of a portion of the prostate in operation for stone, without serious detriment.

Dr. W. F. Westmoreland said he regarded the pathological specimen as a very interesting one. The growth of the prostate was not sufficiently large to be very readily detected by the ordinary means of diagnosis, and proves that retention of urine may occur from this cause when not suspected. Has used large doses of ergot, sometimes with relief, but not very generally. The plan of injecting iodine with hypodermic syringe into the prostate has been practiced, and while reports of cure by this means have been made, the unpleasant and even fatal results which often follow have measurably destroyed confidence in the measure. The crushing and extracting plans have been attempted, but from the imminent danger of such operations, they are not frequently practiced.

Dr. McIntosh: Why are such operations dangerous?

Dr. Westmoreland: Because it is in the old subject that enlargement of the prostate gland occurs, and such cases do not tolerate serious operations. The dangerous results of crushing and abstraction are: hemorrhage, low forms of inflammation, septicæmia, and exhaustion.

Academy adjourned.

---

J. S. TODD. M.D., Reporter.

---

Atlanta, March 20, 1877.

Meeting called to order by the President, Dr. V. H. Taliaferro.

Dr. A. W. Calhoun reported the case of a child now five months old, who at this tender age presented quite a pathological museum. For a few days after birth it had yellow fever—its mother suffering from it when it was born, in Savannah during the recent epidemic in that plague-stricken city. It then had congenital central cataract in one eye and purulent ophthalmia, which was followed by total staphyloma in the other. For the latter affection it came under his observation. Staphyloma is a name given by occulists to different protrusions of the anterior surface of the eye.

The ball in this case is enlarged laterally, and bulges one-third of an inch beyond parallel line drawn from point of greatest projection of other eye. The daily growth forward of eye is quite perceptible. The child at present suffers no pain, but atmospheric influences, dust, etc., acting upon it, is a constant source of irritation, which will continue necessarily under the nature of things, and rendered an operation unavoidable. So he decided to perform Crichett's operation. This differs from enucleation in that in the latter surgical procedure the entire ball is taken out; whereas in the former only about half is amputated, which leaves a stump with all the muscles attached, thereby giving all the motions to an artificial eye that are enjoyed by a natural one, the advantages of which are obvious. For the purpose of doing Crichett's amputation, five curved needles, armed with silk ligatures, are inserted at a point on the ball a little behind the juncture of cornea and sclerotic. When this part thus transfixed is cut, you take away the cornea, iris, lens, ciliary bodies, and also part of vitreous humor; the remainder of this substance being prevented from escaping by the stitches, which are then tied and removed generally about the end of seven or eight days. This operation sometimes fails from pan ophthalmitis supervening; the cavity occupied by the vitreous becomes filled with pus and shrinks when this is evacuated; occasionally inflammation extends along the course of the optic nerve to the meninges of the brain; and this is more apt to occur in children than adults. The case to date is progressing favorably.

Note.—Perfect success has crowned Dr. C.'s efforts in this case.

Dr. Taliaferro detailed an interesting case of complete cystocele, with hypertrophic elongation of neck of the uterus, successfully treated by Schrœder's operation for narrowing the vagina, performed by himself before the college class.

The unfortunate woman was unable to empty the bladder without the introduction of the catheter. The elongation of the cervix uteri caused it to protrude beyond the vulva. She had had severe attacks of cystitis and disturbances consequent thereupon.

The parts were replaced and the woman confined to the prone posture. Daily dressings of wool, soaked in glycerine, were practiced for fourteen days, when it was found that the depth of uterus had diminished from six to three inches, and that all inflammation had been subdued.

The operations for narrowing the vaginal canal are several. The most usual and popular is probably that of Sims and Emmett, which consists in vivifying the edges of a triangular space in the vaginal mucous membrane, the base above, near the cervix uteri, and apex at lower portion. He objects to this operation because the vagina is folded upon itself, and is apt to break loose from the dragging of an enlarged uterus, and is almost sure to do so if the woman gives birth to children; and when this does occur the patient is left in worse condition than before the attempt at cure.

Dr. Thomas uses a glove stretcher, which is inserted through an incision in the mucous membrane, and with it loosens that membrane from the sub-mucous tissues. He then encloses in a clamp such portion of mucous membrane as he desires or deems necessary, and with a suitable instrument shaves this off; but is often foiled by pus forming, etc.

The operation which he performed, and that of which he most approves, consists in taking out an oval piece of the vaginal mucous membrane, which in this case was two inches in its greatest diameter, it being at the centre of the figure. He used a Sims speculum—that indispensable to most all gynæcological procedures—and side retractors, and marked out with ink the portion of membrane which was to be dissected. The woman was not in the usual position preferred by Sims, but on the back (Simons' position). About eighteen stitches were used to bring the mucous surfaces together. They were alternately deep and narrow, i.e., say the first stitch would go from one edge of cut mucous membrane to the other, thus showing all the thread on the dissected surface; the second would penetrate the mucous membrane at the edge of the incision, and the needle be brought out at the middle of dissected space, and again in this space he inserted it, coming up at opposite edge of wound a little distance in mucous membrane. Thus all the thread was hid from view except at point of insertion, exit, and in the middle of dissected surface.

This procedure he thought was the most effectual mode of preventing pus, should any form, from bagging.

The woman could not wear a pessary; so great was the pressure that it cut through the mucous membrane before the operation, but could now do so with comfort; and he has advised that Albert Smith's pessary—which is a modification of Hodge's—be worn indefinitely.

The success in this case was complete; it now being sixty days since the operation. The sutures were removed on the seventh or eighth day after operation. The hemorrhage consequent upon the operation, contrary to his expectation, was slight. Subinvolution was the cause of all the trouble, and this condition, to some extent, is still a factor in the case. The oval piece of membrane was taken from the anterior surface of the vagina, and extended from cervix to near the meatus urinarius.

Academy adjourned.

